# Design and Testing of a Portable Wireless Multi-Node sEMG System for Synchronous Muscle Signal Acquisition and Gesture Recognition

**DOI:** 10.3390/mi16030279

**Published:** 2025-02-27

**Authors:** Xiaoying Zhu, Chaoxin Li, Xiaoman Liu, Yao Tong, Chang Liu, Kai Guo

**Affiliations:** 1 Division of Life Sciences and Medicine, School of Biomedical Engineering (Suzhou), University of Science and Technology of China, Hefei 230026, China; 2 Suzhou Institute of Biomedical Engineering and Technology, Chinese Academy of Sciences, Suzhou 215163, China; 3 Department of Rehabilitation Medicine, The People’s Hospital of Suzhou New District, Suzhou 215011, China; 4 Jinan Guoke Medical Technology Development Co., Ltd., Jinan 250001, China; 5 Chongqing Guoke Medical Innovation Technology Development Co., Ltd., Chongqing 404101, China

**Keywords:** surface electromyography signal, multi-channel, wireless transmission, gesture recognition

## Abstract

Surface electromyography (sEMG) is an important non-invasive method used in muscle function assessment, rehabilitation and human–machine interaction. However, existing commercial devices often lack sufficient channels, making it challenging to simultaneously acquire signals from multiple muscle sites.In this acticle, we design a portable multi-node sEMG acquisition system based on the TCP protocol to overcome the channel limitations of commercial sEMG detection devices. The system employs the STM32L442KCU6 microcontroller as the main control unit, with onboard ADC for analog-to-digital conversion of sEMG signals. Data filtered by analogy filter is transmitted via an ESP8266 WiFi module to the host computer for display and storage. By configuring Bluetooth broadcasting channels, the system can support up to 40 sEMG detection nodes. A gesture recognition algorithm is implemented to identify grasping motions with varying channel configurations. Experimental results demonstrate that with two channels, the Gradient Boosting Decision Tree (GBDT) algorithm achieves a recognition accuracy of 99.4%, effectively detecting grasping motions.

## 1. Introduction

Surface electromyography (sEMG) serves as a crucial tool in the assessment of motor disorders within the field of neuromuscular pathophysiology and finds broad application in disciplines such as neurology [1], neurophysiology [2], orthopedics [3], and rehabilitation [4,5,6]. Its primary applications include the following: performing fundamental measurements under static conditions in conjunction with nerve stimulation to evaluate the direct or reflexive responses of relaxed muscles [7,8,9], thereby complementing conventional needle electromyography and nerve conduction studies; analyzing excitability and signal transmission along specific pathways through peripheral or central stimulation under static or quasi-static conditions, offering pathophysiological diagnostic insights into motor control disorders [10]; and recording muscle activity during dynamic tasks, such as gait analysis [11,12,13,14], to uncover functional and pathological features of abnormal gait patterns. The effective use of sEMG requires careful selection of recording systems, signal acquisition methods, data processing algorithms, and interpretation strategies, tailored to the patient’s age, specific pathology, and diagnostic objectives, to achieve customized and efficient diagnostic outcomes.

The detection of sEMG signals is a complex process influenced by the anatomical structure of muscles, the physiological processes underlying signal generation, as well as external factors and noise. Common noise sources include inherent electronic noise from recording equipment and motion artifacts. The former refers to white noise with frequency components ranging from DC to several kilohertz [15], while the latter consists of noise within the 1–15 Hz range, comparable in amplitude to sEMG signals. Motion artifacts are typically caused by movements at the electrode-skin interface or by cable motion. Additionally, electromagnetic noise, such as 50 Hz or 60 Hz power line radiation, can disrupt sEMG signals. While notch filters are theoretically capable of removing such noise, they may also attenuate other frequency components of the sEMG signals [16]. Therefore, alternative denoising techniques are recommended, like digital signal processing techniques from classical digital filters to modern filtering techniques such as wavelets [17,18,19,20].

A critical issue in the design of sEMG systems is the appropriate design of the biological amplifier. First, the biological amplifier should be compact and lightweight to ensure it can be worn during movement. Second, the bioelectrical amplifier needs to exhibit high gain, low equivalent input noise, a high common-mode rejection ratio (CMRR), and high input impedance [21,22]. Most of these requirements can be met by using a monolithic instrumentation amplifier (IA) as the front-end stage [21]. Since the required gain for an sEMG amplifier is at least 1000, a single-stage amplifier cannot achieve this level of gain due to output saturation issues. Therefore, the gain of the front-end instrumentation amplifier is typically set around 100, with additional gain provided by the second-stage amplifier, usually an operational amplifier. Additionally, the sEMG bioamplifier should be designed as a filter to suppress DC offset and serve as an anti-aliasing filter.

As the electrical activity of adjacent muscles is easily recorded, effectively avoiding such noise remains a significant challenge. Among these, remote transmission monitoring has become the mainstream. Compared to wired transmission, remote transmission offers the advantages of stability and portability. Chang et al. [23] developed a wireless sEMG acquisition system for the detection and assessment of muscle fatigue, utilizing the MSP430-F5438 microcontroller (Texas Instruments, Dallas, TX, USA) as the central processing unit. The system transmits data to a host computer via a Bluetooth module. However, this system is limited to dual-channel signal acquisition and does not support the collection of additional channels. In contrast, Yang et al. [24] proposed a wireless multi-channel sEMG acquisition system that employs WiFi for data transmission and supports up to 30 channels. This system integrates wearable EMG sensors, a microcontroller unit, and a Wi-Fi module to transmit acquired sEMG signal samples to the host for real-time processing. However, it does not address the synchronization issue across multiple channels. Zhu et al. [25] introduced a wearable wireless system designed for multi-channel sEMG detection, which utilizes Bluetooth communication with a host computer and achieves a high sampling rate of 2 kHz. This system incorporates a multi-channel ADC chip for capturing signals from eight channels and employs polling to ensure real-time data transmission and synchronization. Nonetheless, the number of channels is constrained by the capacity of the ADC chip.

Therefore, this paper focuses on portability, multi-node architecture, and stability based on driven-right-leg circuit design. It combines the characteristics of electromyography and employs the STM32 series microcontrollers and ESP8266 WiFi modules from STMicroelectronics (Geneva, Switzerland) to design a remote, portable multi-channel sEMG acquisition system. The system can stably and effectively acquire multi-channel sEMG signals and display them in real time through the host computer through digital filter. Additionally, gesture recognition algorithms are applied to identify gripping actions.

## 2. Materials and Methods

### 2.1. System Overall Design

**Figure 1:**
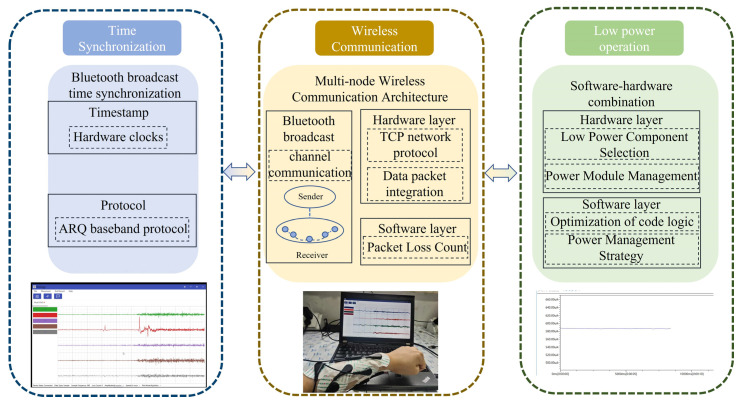
Overview of multi-node sEMG acquisition system.

The overall architecture of the system is shown in Figure 1, which illustrates a multi-node network-based sEMG signal acquisition and transmission system. The system consists of multiple independent sEMG signal acquisition nodes. Each node collects surface electromyographic signals from the target muscle via electrodes. The signals first pass through a preprocessing circuit, which primarily removes high-frequency noise and interference to ensure the quality of subsequent signal processing. After filtering and amplification, the electromyographic signals are sent to the STM32 microcontroller (STMicroelectronics, Geneva, Switzerland) of the node. The built-in 12-bit analog-to-digital converter (ADC) of the microcontroller converts the analog signals into digital signals for further processing and analysis.

The converted digital signals are transmitted via the Serial Peripheral Interface (SPI) to the ESP8266 WiFi module for data transmission. The ESP8266 WiFi module serves as the communication bridge, responsible for wirelessly transmitting the sEMG data from each node to the host computer for real-time processing and monitoring. The system is also equipped with a Si24R1 (Silicon Laboratories, Austin, TX, USA) synchronization module that receives broadcast signals from the synchronizer to ensure that the electromyographic signals from multiple nodes are synchronously acquired and transmitted. This synchronization module transmits the synchronization signals to the WiFi module, ensuring that data from all nodes are uploaded in sync to the host computer.

For power supply, the lithium battery power management module provides stable power to support continuous sEMG signal acquisition. This module includes functions such as charging, discharging protection, and overload protection, ensuring the safety and stability of the system during long-term use. The overall system design provides high efficiency, real-time performance, and reliability, meeting the demands for long-term, multi-node sEMG signal acquisition while efficiently transmitting large volumes of data to the host computer for subsequent analysis and processing.

**Figure 2:**
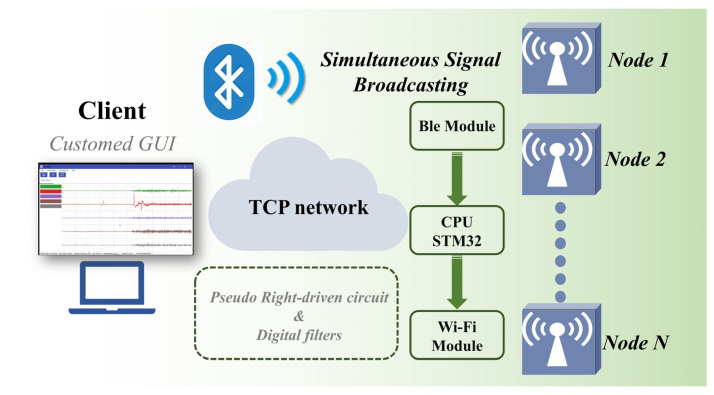
Illustration of system WiFi network.

The WiFi networking and connection are shown in Figure 2, illustrating the wireless communication architecture of the multi-node sEMG data acquisition system. In this system, multiple nodes are responsible for acquiring sEMG signals. Each node’s signal is first processed through the preprocessing circuit for filtering and noise reduction to ensure data quality. Each node receives synchronization signals to ensure the temporal alignment of data collection, which improves the overall consistency and accuracy of the data. The synchronization signals are transmitted by a dedicated synchronization module. After receiving the synchronization signal, all nodes start collecting sEMG signals according to the unified time reference, ensuring that the data acquired by each node is synchronized for processing. The synchronized data from each node is wirelessly transmitted through the ESP8266 WiFi module via a TCP network to the client host computer for real-time processing.

The WiFi network design enables flexible and efficient data transmission from multiple nodes, making it especially suitable for sEMG signal processing applications that require efficient synchronization and multi-channel data collection. With this design, multiple distributed nodes can collaborate seamlessly, transmitting electromyographic signals in real time and with high accuracy to the host computer for further analysis and processing.

### 2.2. System Hardware Design

The hardware system consists of two main parts: the synchronizer and the single-node acquisition circuit board. The core of the synchronizer is the STM32L452KCU6 microcontroller (STMicroelectronics, Geneva, Switzerland), which is responsible for coordinating the synchronization operations across the entire system. Specifically, the STM32L452KCU6 (STMicroelectronics, Geneva, Switzerland) controls the Si24R1 module to broadcast synchronization signals to all nodes, ensuring that the nodes acquire electromyographic data based on a unified time reference. This synchronization mechanism is crucial as it ensures the temporal consistency of the data between multiple nodes, preventing misalignment or data confusion, thereby providing a reliable foundation for subsequent data analysis and processing.

**Figure 3:**
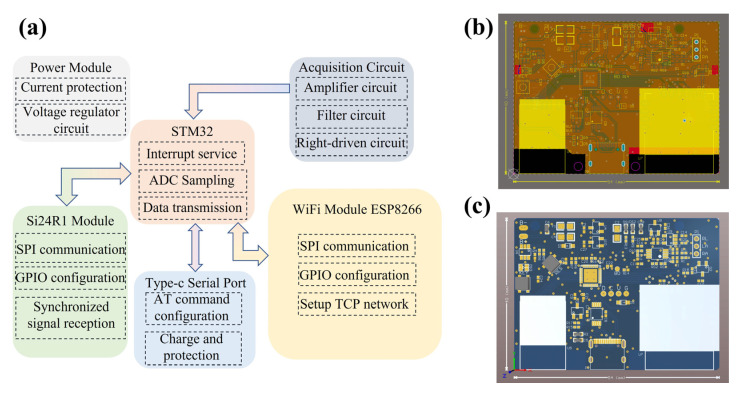
Illustration of single-node acquisition circuit board. (**a**) Schematic diagram of the single-node acquisition circuit board. (**b**) Two-dimensional view of the single-node acquisition circuit board. (**c**) Three-dimensional view of the single-node acquisition circuit board.

The framework of the single-node acquisition circuit board is shown in Figure 3. Each node is equipped with multiple modules to ensure the proper operation of the system and the precise acquisition of data. First, each node is equipped with a filtering and amplification module that processes the raw signals from the sEMG sensors (3M, St. Paul, MN, USA). The filtering and amplification module effectively removes noise components from the sEMG signals and amplifies the signals to prepare them for subsequent digitization. The circuit board also includes a synchronization signal reception module that receives the synchronization signals from the synchronizer, ensuring that the node’s data acquisition is in sync with the other nodes. This module’s primary function is to ensure that each node, upon receiving the synchronization signal, begins acquiring electromyographic data at the same time, thus enabling multi-node synchronized data collection. Additionally, each node is equipped with a WiFi module to wirelessly transmit the acquired data to the host computer. The WiFi module communicates with other nodes over a local area network (LAN) and transmits the synchronized sEMG signals to the host computer for further processing and analysis. This wireless communication module not only provides flexible data transmission but also enhances the system’s scalability, allowing for the easy addition of more nodes to the network.

#### 2.2.1. sEMG Signal Preprocessing Circuit

**Figure 4:**
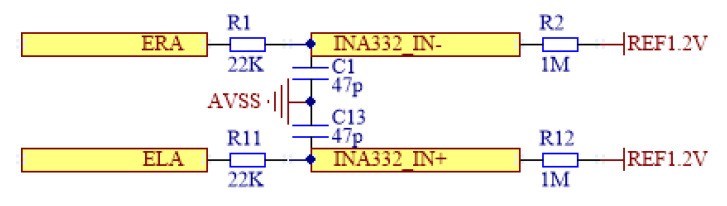
Pre-processing circuit.

The sEMG signal preprocessing circuit, shown in Figure 4, is designed based on the characteristics of sEMG signals. It uses a direct current (DC) coupling circuit to collect the sEMG signals. DC coupling avoids the filtering of high-frequency signals, enabling the signal to cover a wide range from resting potential to active muscle potentials, thus improving the signal capture accuracy. The collected sEMG signal is a differential signal, which is processed by the amplification module before being sent to the STM32 microcontroller (STMicroelectronics, Geneva, Switzerland) for further processing.

**Figure 5:**
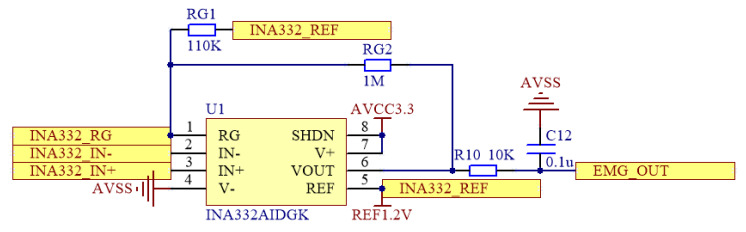
Signal amplifier circuit.

In the amplification circuit, the INA332 (DGK) chip (Texas Instruments, Dallas, TX, USA) is selected as the amplifier as shown in Figure 5. This chip is known for its high precision, low noise, and low power consumption, making it suitable for amplifying sEMG signals. By adjusting the feedback resistor values, the amplification factor can be flexibly configured. In this design, the amplification factor is precisely adjusted to achieve a gain of 96×, which not only enhances the signal amplitude but also effectively improves the signal-to-noise ratio (SNR) of the weak sEMG signals in the subsequent processing stages.

To further optimize signal quality, an RC passive filter circuit is designed in the system. The primary purpose of this circuit is to filter the sEMG signal’s frequency components, removing high-frequency noise and unnecessary frequency bands. Specifically, the cutoff frequency of the low-pass RC filter is set at 159.2 Hz, which effectively filters out high-frequency noise above this threshold while preserving the frequency range relevant to muscle activity, ensuring the signal’s validity and accuracy.

#### 2.2.2. Pseudo Right Leg Shielding Drive Circuit

The right leg drive circuit is commonly added to biological signal amplifiers to reduce common-mode interference. Biological signal amplifiers, such as those used for electrocardiograms (ECG), electroencephalograms (EEG), or sEMG, measure the very small electrical signals emitted by the body [26]. However, the human body can also act as an antenna for electromagnetic interference, particularly from power lines at 50/60 Hz. This interference can obscure biological signals, making them difficult to measure. The right leg drive circuit is used to actively cancel out interference and reduce noise [27].

**Figure 6:**
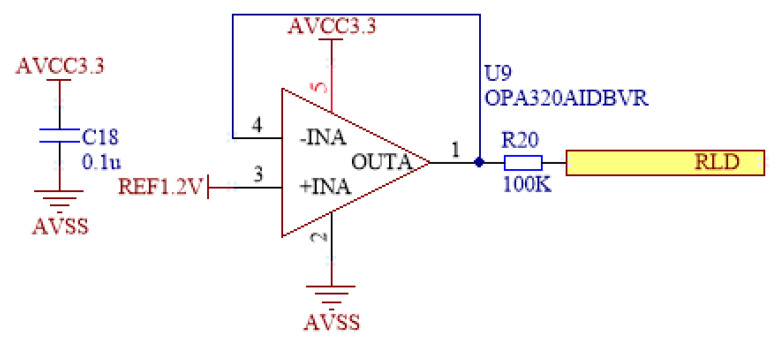
Pseudo right-driven circuit.

Thus, we propose the pseudo right-leg drive circuit shown in Figure 6, consisting of a low-noise operational amplifier (OPA320 (Texas Instruments, Dallas, TX, USA)). This circuit actively drives the body using a known voltage source to prevent electrical floating during the measurement process. When the body is capacitively coupled away from the reference voltage, the right-leg drive amplifier compensates for this by pulling the body potential back to the set value, thereby maintaining the voltage balance between the body and the amplifier. This active driving mechanism ensures that the common-mode voltage of the signal remains within a stable range, avoiding signal distortion due to unstable potentials. In this design, a voltage of 1.2 V is applied to maintain the common-mode voltage within a specified range, which effectively suppresses common-mode interference from environmental noise (such as power line interference), and serves as an analog ground for the pre-common-mode amplifier circuit while improving the accuracy of biological signal measurements. The key aspect of this circuit design is its low noise characteristics and high stability, providing effective interference suppression, especially in high-noise environments, and ensuring reliable biological signal acquisition and processing.

#### 2.2.3. Processor Control Section

**Figure 7:**
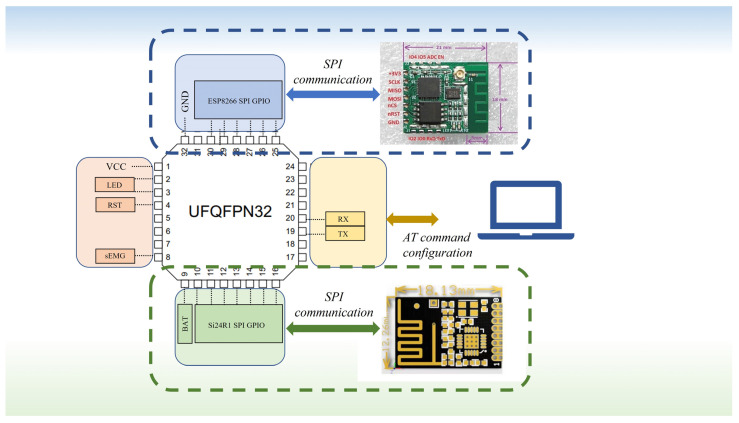
Structure of STM32 CPU peripheral interface.

The system uses the STM32L442KCU6 microcontroller for the control section. This chip operates with a low voltage supply of 1.71–3.6 V and features 26 fast I/O ports. It operates at a frequency of up to 80 MHz and includes high-speed Flash and RAM memory. It also has 2 SPI interfaces, 2 I2C interfaces, 3 USART interfaces, and 1 LPUART interface. This meets the design requirements while maintaining a compact size. The peripheral interface circuit is shown in Figure 7. The processor’s functions include: using the SPI communication interface to transmit signals to the WiFi and synchronization modules, performing ADC, generating timestamps using a clock, converting battery voltage, and controlling the power-on/power-off LED display.

#### 2.2.4. Power Management Module

**Figure 8:**
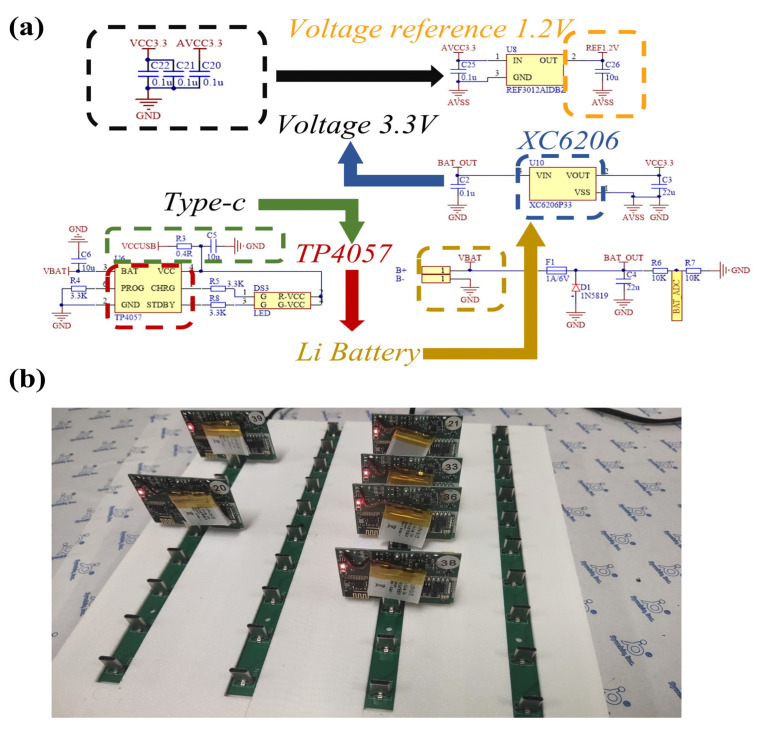
Power management structure and charging. (**a**) Power circuit and structure. (**b**) Array charging display.

The power management and charging module, shown in Figure 8, is designed for long-term operation and portability. The system is powered by a single 4.2 V lithium battery, which is protected by a fuse and diode. The TP4057 chip (Tianjin Lishen Battery Joint-Stock Co., Ltd., Tianjin, China) is used to manage the charging of the lithium battery, featuring charge and full-charge indicator lights. The charging voltage is fixed at 4.2 V, and once the battery reaches this voltage, the entire circuit enters a low-power state, reducing the chip’s static current to a tenth of its original value. The chip includes reverse-polarity protection and anti-reverse charging circuitry, with the charging current adjustable by an external resistor, set to 300 mA in this system. The chip also has thermal feedback to automatically adjust the charging current to limit chip temperature under high-power operation or in high-temperature environments. To meet the voltage requirements of the system’s components, the XC6206 (Torex Semiconductor Ltd., Tokyo, Japan) linear voltage regulator is used to convert the 4.2 V battery voltage to a 3.3 V supply for the system’s chips. Battery voltage is sampled by the internal ADC of the STM32 processor (STMicroelectronics, Geneva, Switzerland) to monitor the remaining battery power.

### 2.3. System Software Design

**Figure 9:**
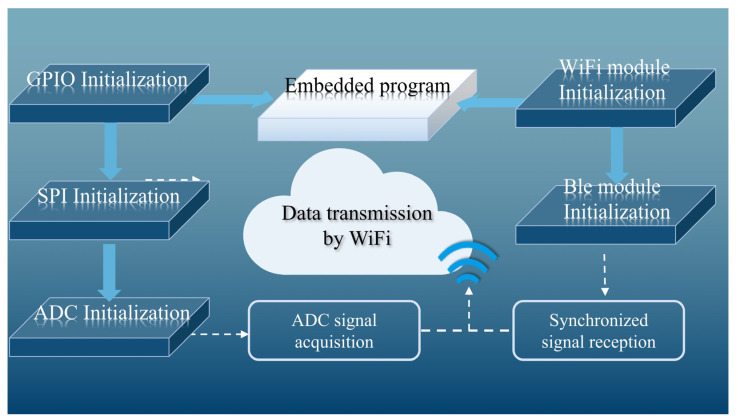
Illustration of sEMG acquisition single-node board embedded software.

The program structure of the STM32L442KCU6 (STMicroelectronics, Geneva, Switzerland) is shown in the figure below (Figure 9), which consists of four core modules: the ADC module, the data buffering module, the synchronization signal detection module, and the WiFi transmission module. The entire system relies on the cooperation of these modules to achieve the functionality of collecting sEMG signals and wirelessly transmitting the data to the host computer.

#### 2.3.1. ADC Module

The ADC module built into the STM32L442KCU6 microcontroller offers high-precision ADC capabilities. In this system, the ADC module is configured in channel scanning mode, with a data format set to 12-bit right alignment, ensuring sufficient precision when acquiring sEMG signals. The single conversion mode, combined with the activation of the Direct Memory Access (DMA) channel, allows for efficient data transfer during the acquisition process, reducing the burden on the CPU. To ensure sampling accuracy and real-time performance, the system is also configured with a 2 ms clock source triggering the TIM1 PWM event, which ensures that the ADC can perform data acquisition at appropriate time intervals. In the interrupt service function, the system uses DMA to transfer the ADC-acquired data to the FIFO queue, enabling real-time data acquisition of sEMG signals.

#### 2.3.2. Data Buffering Module

The memory of the STM32L4 series microcontroller is divided into 48 KB of SRAM1 and 16 KB of SRAM2. To improve the efficiency and flexibility of the system’s data buffering, sufficient memory space is first requested during the initialization of the data buffer module. If the memory allocation fails, the system will release any previously allocated space to prevent memory leaks. The data buffer adopts a circular queue structure, which includes fields for data status, transport count, queue capacity, and a queue clearing flag. This structure is used to store information about the current queue and can dynamically request contiguous queue space, ensuring maximum memory utilization and efficient data transfer. This design not only ensures efficient data access but also improves the stability and reliability of the system under different operational conditions.

#### 2.3.3. Synchronization Signal Detection Module

The reception and processing of the synchronization signal are carried out by the Si24R1 module, which is responsible for receiving the broadcast synchronization signal sent by the synchronizer. The frequency of the synchronization signal is set to 1 Hz, and the broadcast data packet includes a checksum for validation and a data frame number. When the broadcast signal is received, the IRQ pin of the Si24R1 module triggers an interrupt signal, invoking the interrupt handling function in the main microcontroller. Within this function, the system acquires and transmits the data frame number and synchronizes the start of the TIM2 clock soft interrupt, which begins the sEMG data sampling process. Each time the TIM2 interrupt occurs, the system updates the data frame within the sEMG data packet, ensuring that the data acquisition process is strictly aligned with the synchronization signal, thereby ensuring the synchronization of data across multiple nodes.

#### 2.3.4. WiFi Transmission Module

**Figure 10:**
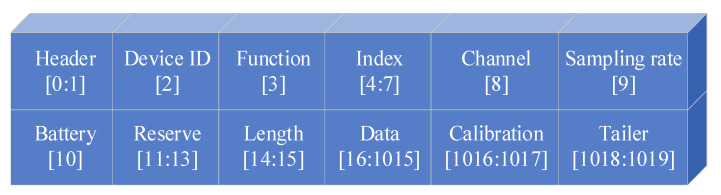
Design of data protocol.

The WiFi module is based on the ESP8266 chip and is configured with network parameters through serial communication using AT commands to complete the device’s networking process. By establishing a TCP communication channel, the system achieves transparent data transmission, enabling real-time transmission of the acquired sEMG signals to the host computer for processing. The data protocol design is illustrated in Figure 10, where the device ID is configured via serial AT commands to allow the host computer to identify different devices and display the sEMG signals from different channels. The checksum in the data packet is calculated using a byte accumulation method, with the high byte first, to ensure the integrity and accuracy of data transmission. The packet sequence number corresponds to the data frame number from the synchronizer, forming a cyclic mechanism that effectively tracks packet loss rates and performs error detection. In the main control program, all collected data packets are formatted in a specific way and transmitted to the ESP8266 module via SPI communication, completing the wireless transmission of the data.

### 2.4. System Testing

#### 2.4.1. sEMG Collection Test

**Figure 11:**
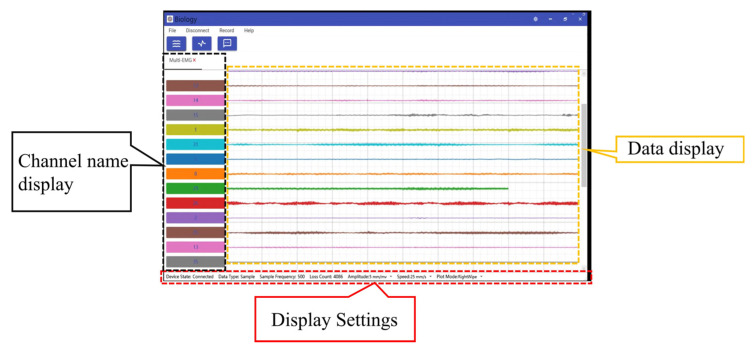
Upper computer display with multi-node sEMG devices.

Under natural room temperature conditions, with the subject in good health and relaxed, the sEMG collection system was tested. AgCl electrode patches were used for signal acquisition, and the electrodes were placed according to the muscle arrangement. The signals were transmitted to the collection system via lead wires. The system’s host computer functions include data reception and analysis, data storage and management, data visualization, and data processing. Figure 11 shows the host system’s reception screen, which allows real-time viewing of sEMG signals from different channels while simultaneously monitoring the packet loss rate. After applying a 50 Hz powerline interference filter and band-pass filtering, the system effectively removed noise, and the sEMG signals were clear.

To assess the performance of the sEMG acquisition system under dynamic conditions, we conducted tests involving motion-induced artifacts and external noise. These tests were performed during treadmill running and indoor walking, focusing on the gastrocnemius muscle signals of the lower leg. This setup was chosen to evaluate the system’s capability in handling the challenges presented by motion artifacts and environmental noise, which are common in dynamic sEMG data collection.

**Figure 12:**
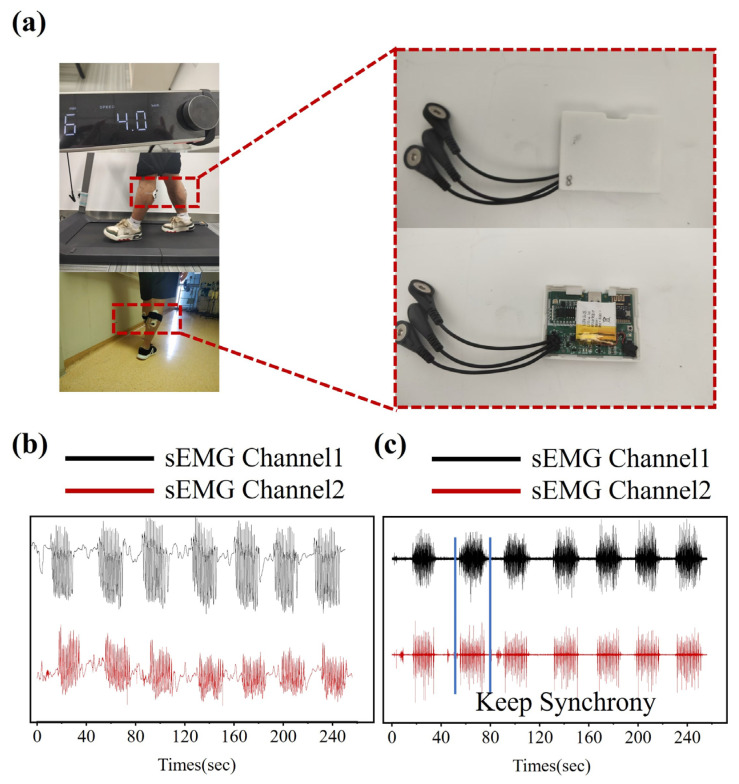
sEMG Signal Acquisition and Processing under Dynamic Conditions. (**a**) Signal Acquisition Board with 3D-Printed Enclosure Used in Walking Experiment. (**b**) Raw sEMG Signals Collected during Walking. (**c**) Filtered sEMG Signals after Digital Filtering.

To enhance user comfort and minimize noise interference, a 3D-printed enclosure made from resin material was designed for the single-node acquisition board, as shown in Figure 12a. This innovative design not only improves the comfort of the wearer but also provides noise isolation, contributing to more reliable signal acquisition.

Figure 12b presents the raw sEMG signals recorded during walking, which exhibit motion artifacts typically caused by muscle movement and external factors. However, through the application of digital filtering techniques, as demonstrated in Figure 12c, these artifacts were effectively eliminated, yielding clear and accurate sEMG signals that meet the criteria for further processing and analysis.

#### 2.4.2. Parameter of the System

To validate the performance of the system, we conducted power consumption tests. Under normal operation, the average current consumption for a single node in standby and power-on states was measured as 29.89 mA, and the average current consumption during active operation was 35.75 mA. The average current consumption of the WiFi module was 586.88 µA. Under normal working conditions, a single node can operate continuously for 6 h, which meets the requirements for sEMG testing.

**Figure 13:**
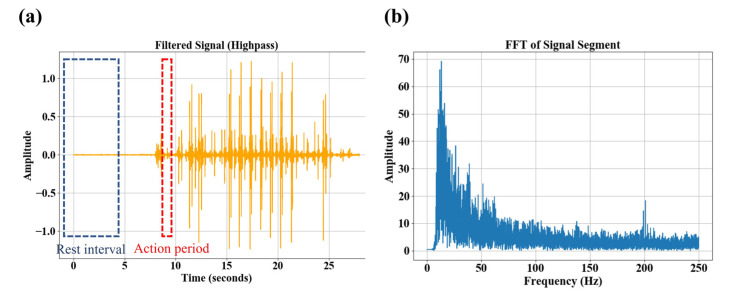
Results of sEMG acquisition and analysis. (**a**) The real-time waveform of sEMG during walk. (**b**) The frequency spectrum.

Furthermore, we evaluated the signal-to-noise ratio (SNR) of the system. By extracting electromyographic signals during motion and at rest, the system’s SNR was calculated to be 24.6 dB (under motion conditions), as shown in Figure 13a. This result indicates that the system achieves high signal quality during sEMG signal acquisition. To further analyze the frequency characteristics of the signal, we performed a Fast Fourier Transform (FFT) on the electromyographic signals. The results revealed that the signal energy is primarily concentrated within the range of 10 Hz to 150 Hz, as illustrated in Figure 13b. This frequency distribution aligns with the 159.6 Hz cutoff frequency of the analog filter on the system’s circuit board, demonstrating the system’s capability to effectively capture the main energy distribution range of sEMG signals.

Other key parameters of the system are summarized in Table 1. The system supports synchronous acquisition across 40 channels, with a sampling frequency of 500 Hz, an A/D resolution of 12 bits, a bandwidth of 10 Hz to 150 Hz, and a gain of 96. Communication is facilitated via WiFi, with a PC as the receiver. The device dimensions are 5.4 cm × 4 cm × 2 cm, with a weight of 31.7 g, and the electrode material is Ag/AgCl. These parameters ensure high precision and portability in sEMG signal acquisition.

Moreover, to benchmark the proposed system against commercial solutions, we summarize key specifications in Table 2. While our system achieves a moderate usage time of 6 h (surpassing FREEMG300 (OT Bioelettronica, Salerno, Italy) [28] and Due+Pro (OT Bioelettronica, Salerno, Italy) [29], but shorter than Ultium EMG (Delsys Inc., Natick, MA, USA) [29] and NeuroHUB (Neurorehab Technologies, Toronto, ON, Canada) [30]), it uniquely balances high channel density and wireless efficiency. The 12-bit ADC and 500 Hz sampling rate strike a balance between performance and cost, sufficient for sEMG signal acquisition, with 40 channels, our system outperforms all listed commercial devices (16 channels), enabling comprehensive multi-muscle monitoring for complex tasks like full-arm gesture analysis. Despite higher power consumption compared to BLE, WiFi ensures high-speed data transmission, while maintaining competitive usage time.

### 2.5. Gesture Recognition Based on sEMG

Gesture recognition holds significant potential for prosthetic control and offers promising capabilities for the development of real-time systems detecting hand grasping movements. Palermo1 et al. [31]. had participants perform various grasping actions to grab objects and recorded the sEMG from the forearm muscles. They used a random forest classifier to compare the classification accuracy of data from different times and different participants, in order to evaluate the repeatability of the sEMG data. The results showed that when training and testing the classifier with data collected at different times, the classification accuracy decreased by an average of 27.03%, but was still significantly higher than random chance, indicating that data collected at different times can still be used for training classification algorithms. Ouyang et al. [32]. designed four distinct hand grasping tasks and recorded sEMG signals from the forearm muscles. They employed both linear and non-linear measures to effectively analyze the dynamic characteristics of sEMG signals during the hand grasping movements, which led to an improvement in classification accuracy.

Building on these methods, our study aims to explore gesture recognition using varying numbers of electrodes (1, 2, and 4 channels) for sEMG signal acquisition, with a particular focus on the forearm muscle groups primarily responsible for controlling grasping gestures. Additionally, we compare the performance of different machine learning algorithms through feature extraction to evaluate their classification effectiveness.

#### 2.5.1. Experimental Design

**Figure 14:**
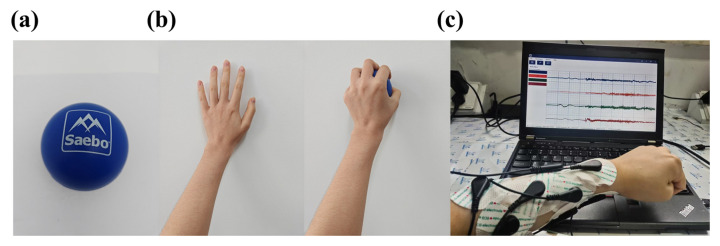
Experimental setup for grasping task and sEMG acquisition. (**a**) Silicone ball used as the grasping object. (**b**) Hand gestures of relax and grasp. (**c**) Real-time acquisition of sEMG.

The experiment involved the collection of sEMG data from four participants who had no previous history of neurological or muscular disorders, each performing grasping ac-tions with a single arm using devices with different numbers of electrodes (1, 2, and 4 channels). We use Saebo Balls (Saebo, Inc., Charlotte, NC, USA) as the target object for grip tasks, as depicted in Figure 14a. The ball is constructed from silicone, with approximate dimensions of 21.59 × 27.31 × 9.53 cm and a weight of 18.9 g. Designed specifically for rehabilitation purposes, this device is particularly suitable for the present study. During the experiment, each participant was instructed to grasp the silicone ball with moderate force, as illustrated in Figure 14b. To minimize the impact of muscle fatigue on sEMG signal collection, participants were instructed not to perform any bilateral muscle exercises for three days prior to the experiment. Each grasping action was repeated 10 times in a group, with each action lasting 5 s, followed by 5 s of rest. To avoid muscle fatigue, participants performed only one set of experiments per day, ensuring consistent conditions across all stages. Participants were also instructed to follow the same procedure in each stage. During rest periods, participants relaxed their arms, placing them flat on their legs or on a table. The target skin surface is cleaned with alcohol wipes to remove excess dust, dead skin cells, and skin oils that may interfere with sEMG signals before data acquisition. Since grasping gestures are primarily associated with finger movements and are functionally controlled by the forearm muscle groups [33], the commercial Ag/AgCl wet electrodes are used as sensors and are attached to the forearm muscle group. For single-channel acquisition, the electrode was placed on the flexor digitorum profundus. For dual-channel acquisition, an additional electrode was added on the flexor carpi radialis. In the case of four-channel acquisition, electrodes were further placed on the brachioradialis and flexor carpi ulnaris. To validate the robustness of the system algorithm, the electrodes were positioned approximately over the muscle groups rather than at fixed, precise locations. A total of 1200 action segments were obtained, including 400 single-channel action segments, 400 dual-channel action segments, and 400 four-channel action segments.

#### 2.5.2. sEMG Preprocessing and Feature Extraction

**Figure 15:**
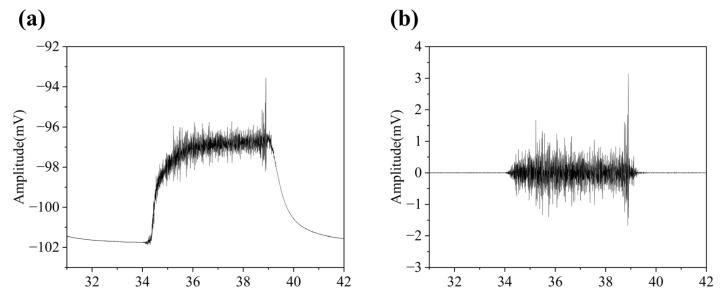
sEMG signals for grasp gesture (**a**) sEMG original signals. (**b**) Filtered sEMG signals through digital filters.

Due to various factors such as the sEMG acquisition equipment, experimental environment, and individual differences among participants, the collected sEMG signals required denoising [34]. A 50 Hz notch filter and a 10–250 Hz band-pass filter were applied to the collected sEMG signals for preprocessing [35]. A comparison of the signals before and after digital filtering is shown in Figure 15. Considering the real-time requirements of gesture recognition and the simplicity of calculating time-domain features [36], 12 features were selected for classification, including: peak value, mean value, average amplitude, root mean square, root mean square amplitude, skewness, kurtosis, waveform index, pulse index, peak index, kurtosis index, and absolute power [37,38,39].

## 3. Results

Seven classification algorithms were used in this study: AdaBoost, Bagging, ExtraTrees, Support Vector Machine (SVM), Random Forest (RF), Decision Forest (DF), and Gradient Boosting Decision Tree (GBDT). The model performance was evaluated based on accuracy, precision, recall, F1 score, and confusion matrix. The true positive (TP), false negative (FN), true negative (TN), and false positive (FP) were calculated by comparing the predicted labels with the actual labels. The formulas for the other four evaluation metrics are given in Equations (1)–(4):

(1) $$Accuracy=\frac{TP+TN}{TP+TN+FP+FN}$$

(2) $$Precision=\frac{TP}{TP+FP}$$

(3) $$Recall=\frac{TP}{TP+FN}$$

(4) $$F1Score=2\times \frac{Precision\times Recall}{Precision+Recall}=\frac{2TP}{2TP+FP+FN}$$

For channels 1, 2 and 4 the classification results are shown below:

From Table 3, it can be observed that the single-channel accuracy is relatively low, with the best-performing classifiers being Bagging, RF, and DF, all achieving an accuracy of 81%. For two-channel data, the performance of all classifiers improves significantly, with overall accuracy exceeding 95%. The best-performing classifier is GBDT, reaching an accuracy of up to 99%. For four-channel data, the performance shows a slight decline compared to the two-channel setup, though it still surpasses that of the single-channel setup. This decline may be attributed to the increased redundancy in the data.

Among the algorithms tested, the best classification results were achieved using data from two channels and the GBDT algorithm, which achieved accuracy and precision rates exceeding 99%.

**Figure 16:**
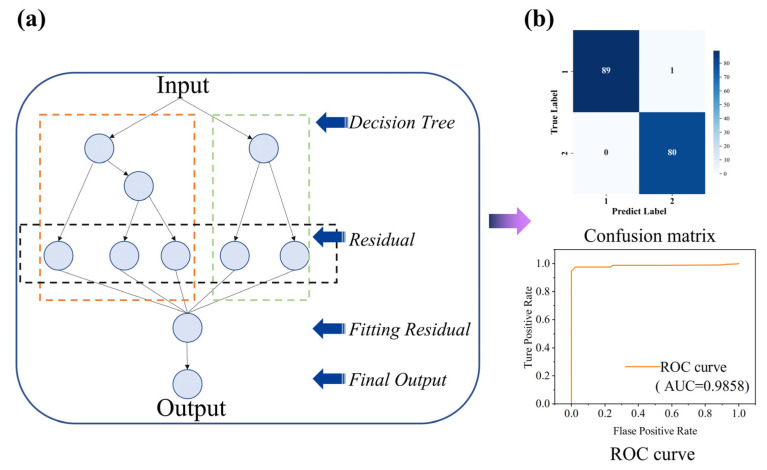
Illustration of GBDT algorithm and resultant diagram of confusion matrix. (**a**) Illustration of GBDT algorithm. (**b**) Two-node grasp gesture GBDT confusion matrix and ROC curve.

GBDT is a Boosting method where multiple weak learners (typically small decision trees) are combined to form a strong learner. Unlike traditional Random Forest (RF), GBDT optimizes the model iteratively. The goal of each iteration is to correct the errors made by the previous model. The training steps are as follows: initialization, calculation of residuals, fitting a new decision tree, updating the model, repeating the iterations, and final prediction. The GBDT flowchart, confusion matrix and receiver operating characteristic (ROC) curve are shown in Figure 16.

## 4. Discussion

This study demonstrates the capability to synchronously acquire signals related to muscle activity. The sEMG acquisition system designed in this study is based on a TCP network and employs DC coupling and ADC counting methods. Using the STM32 series chips from STMicroelectronics (STMicroelectronics, Geneva, Switzerland) and the ESP8266 WiFi module, the system enables multi-node EMG signal acquisition, allowing for the synchronous collection of signals from different muscle groups. The system achieves reliable signal acquisition through the custom-designed right leg drive circuit and the built-in filtering functionality of the host computer. Powered by a lithium battery, the system includes a comprehensive charging, discharging, and protection scheme, enhancing its safety and portability. However, the hardware design, particularly the sensors, lacks comfort when worn. In future work, we aim to address this issue by focusing on the flexibility of the circuitry and improving the sensor design, ensuring the system conforms more closely to the human body. This will enhance wearability and improve the biocompatibility between the sensors and the human body.

In this study, the GBDT algorithm achieved an accuracy of 99.4% using two-channel data. However, due to the small dataset, the model faces the risk of overfitting, and its generalization to new users remains uncertain. Specifically, while the model performs well on the training data, it may not maintain the same performance with new users or in different environments. To address this issue and reduce the risk of overfitting, five-fold cross-validation was used during model evaluation. This approach helped to assess the model’s performance on multiple subsets of the data, ensuring that the model was not overly dependent on a single training set and improving its generalization capability.

To further enhance the model’s robustness, we plan to take the following measures in future work. First, we will expand the dataset, particularly by incorporating data from different user groups (such as varying ages, genders, and health conditions) to enhance the model’s generalization ability. Second, we will increase the diversity of the data by simulating different usage scenarios and dynamic environments to test the model’s stability in real-world applications. Additionally, long-term monitoring and cross-domain experiments will be key focuses of our future work, where we plan to apply the system in clinical settings to validate its stability and adaptability in practical applications.

Furthermore, the commercialization potential of this sEMG system is significant, particularly in the fields of medical rehabilitation, prosthetics, and human–computer interaction. The system’s portability, multi-node architecture, and high accuracy in gesture recognition make it a promising candidate for integration into wearable devices and smart prosthetics. However, several challenges must be addressed before widespread clinical deployment. Firstly, the system’s current design may require further miniaturization and ergonomic improvements to enhance user comfort, especially for long-term use. Secondly, the system’s robustness in diverse clinical environments, such as hospitals or rehabilitation centers, needs to be validated. Factors such as electromagnetic interference, varying skin conditions, and patient movement artifacts could affect signal quality and system performance.

Regulatory considerations also play a crucial role in the deployment of medical devices. The sEMG system must comply with international standards for medical devices, such as the FDA (Food and Drug Administration) regulations in the United States or the CE marking requirements in the European Union. These regulations ensure that the device is safe, effective, and reliable for clinical use. Additionally, data privacy and security are critical, especially when dealing with sensitive patient information. The system must incorporate robust data encryption and secure transmission protocols to protect patient data during wireless communication.

In conclusion, while the system shows great promise, further research and development are needed to address these commercialization and clinical deployment challenges. Collaboration with medical device manufacturers, regulatory bodies, and healthcare providers will be essential to bring this technology from the laboratory to real-world applications.

## 5. Conclusions

This study presents a comprehensive approach to the acquisition, preprocessing, and classification of sEMG signals for gesture recognition. The developed system integrates multiple hardware components, including a synchronizer for multi-node data collection, a signal acquisition circuit for high-quality sEMG signal capture, and a power-efficient management module, ensuring reliable and continuous operation. The acquisition circuit is encapsulated using 3D printing technology, ensuring both durability and a certain level of comfort for the user during prolonged use. The experiment involved the collection of sEMG data from four participants performing grasping actions with different electrode configurations. Among the algorithms evaluated, the Gradient Boosting Decision Tree (GBDT) model demonstrated the best performance, achieving over 99.4% accuracy and precision using data from a two-channel setup. In conclusion, this research highlights the effectiveness of combining advanced signal processing techniques with powerful machine learning models to enhance the accuracy and efficiency of sEMG-based gesture recognition.

## Figures and Tables

**Table 1:** Characteristics of the system.

Description	Value
Numbers of channels	40
Sampling Frequency	500 Hz
A/D Resolution	12 bits
Bandwidth	10 Hz–150 Hz
Gain	96
Signal-to-noise Ratio	24.6 dB (Dynamic)
Communication Type	WiFi
Receiver Type	PC
Usage Time	6
Dimensions	5.4 cm × 4 cm × 2 cm
Weight	31.7 g
Electrode Material	Ag/Agcl

**Table 2:** Comparison with commercial devices.

Systems	Channels	Data Transmission	ADC Resolution (bits)	Sampling Frequency (Hz)	Uasage Time (h)
This papre	40	WiFi	12	500	6
FREEMG	20	BLE	16	1000	5
Due+Pro	16	WiFi	16	500/2000	2.3
Ultium EMG	32	BLE	24	2000/4000	8
NeuroHUB	16	BLE/WiFi	24	4000	14

**Table 3:** Experimental results with different algorithms and number of channels.

Channel	Module	Accuracy	Precision	F1 Score	Recall
1	AdaBoost	80	80.42082	80.03326	80
	Bagging	81.17647	81.31864	81.20261	81.17647
	ExtraTrees	78.82353	78.88072	78.8412	78.82353
	SVC	71.17647	71.28687	71.20952	71.17647
	RF	81.17647	81.59859	81.20777	81.17647
	DF	81.17647	81.23039	81.19218	81.17647
	GBDT	72.94118	73.09704	72.97876	72.94118
2	AdaBoost	97.64706	97.7591	97.64902	97.64706
	Bagging	97.64706	97.67673	97.64836	97.64706
	ExtraTrees	97.64706	97.7591	97.64902	97.64706
	SVC	95.29412	95.53938	95.29803	95.29412
	RF	97.64706	97.7591	97.64902	97.64706
	DF	98.23529	98.29908	98.23658	98.23529
	GBDT	99.41176	99.41903	99.41195	99.41176
4	AdaBoost	91.81287	91.84135	91.81455	91.81287
	Bagging	91.81287	91.84135	91.81455	91.81287
	ExtraTrees	91.22807	91.28783	91.22987	91.22807
	SVC	85.38012	86.15863	85.26181	85.38012
	RF	91.81287	91.84135	91.81455	91.81287
	DF	90.64327	90.64327	90.64327	90.64327
	GBDT	90.05848	90.09776	90.0503	90.05848

## Data Availability

The data presented in this study are available on request from the corresponding author.
